# Development of Olive Oil and α-Tocopherol Containing Emulsions Stabilized by FucoPol: Rheological and Textural Analyses

**DOI:** 10.3390/polym14122349

**Published:** 2022-06-09

**Authors:** Sílvia Baptista, João R. Pereira, Cátia V. Gil, Cristiana A. V. Torres, Maria A. M. Reis, Filomena Freitas

**Affiliations:** 1 Associate Laboratory i4HB—Institute for Health and Bioeconomy, School of Science and Technology, NOVA University Lisbon, 2829-516 Caparica, Portugal; silvia.baptista@73100.pt (S.B.); jra.pereira@campus.fct.unl.pt (J.R.P.); cv.gil@campus.fct.unl.pt (C.V.G.); c.torres@fct.unl.pt (C.A.V.T.); amr@fct.unl.pt (M.A.M.R.); 2 UCIBIO—Applied Molecular Biosciences Unit, Department of Chemistry, School of Science and Technology, NOVA University Lisbon, 2819-516 Caparica, Portugal; 3 73100, Lda. Edifício Arcis, Rua Ivone Silva, 6, 4° Piso, 1050-124 Lisboa, Portugal

**Keywords:** polysaccharide, FucoPol, response surface methodology, oil-in-water emulsions, rheology, texture

## Abstract

Biobased raw materials like natural polysaccharides are increasingly sought by the cosmetic industry for their valuable properties. Such biodegradable and usually non-cytotoxic biopolymers are commonly used in skin-care products as rheological modifiers, bioemulsifiers and/or bioactive ingredients. FucoPol is a natural polysaccharide with reported biocompatibility, emulsion-forming and stabilizing capacity, shear-thinning behavior and bioactivity (e.g., antioxidant capacity, wound healing ability) that potentiate its utilization in skin-care products. In this study, olive oil and α-tocopherol containing emulsions were stabilized with FucoPol. Although the presence of α-tocopherol negatively impacted the emulsions’ stability, it increased their emulsification index (EI). Moreover, FucoPol outperformed the commercial emulsifier Sepigel^®^ 305, under the tested conditions, with higher EI and higher stability under storage for 30 days. The formulation of FucoPol-based emulsions with olive oil and α-tocopherol was studied by Response Surface Methodology (RSM) that allowed the definition of the ingredients’ content to attain high emulsification. The RSM model established that α-tocopherol concentration had no significant impact on the EI within the tested ranges, with optimal emulsification for FucoPol concentration in the range 0.7–1.2 wt.% and olive oil contents of 20–30 wt.%. Formulations with 25 wt.% olive oil and either 0.5 or 2.0 wt.% α-tocopherol were emulsified with 1.0 wt.% or 0.7 wt.% FucoPol, respectively, resulting in oil-in-water (O/W) emulsions. The emulsions had similar shear-thinning behavior, but the formulation with higher FucoPol content displayed higher apparent viscosity, higher consistency, as well as higher firmness, adhesiveness and cohesiveness, but lower spreadability. These findings show FucoPol’s high performance as an emulsifier for olive oil/α-tocopherol, which are supported by an effective impact on the physicochemical and structural characteristics of the emulsions. Hence, this natural polysaccharide is a potential alternative to other emulsifiers.

## 1. Introduction

The cosmetics industry’s interest in moving towards sustainability has significantly increased the incorporation of natural polymers into cosmetic formulations. Among those, many polysaccharides have properties similar to non-biodegradable synthetic polymers, which makes them environmentally friendly alternative raw materials [1,2]. Specifically, bacterial polysaccharides can be used in formulations as moisturizing agents, thickeners, stabilizers and texturizers, acting as a biocompatible and biodegradable component that protect and maintain the skin and improves the formulations efficacy [2,3]. Emulsions, usually used in the cosmetic, pharmaceutical and food industries, are the most common type of skincare products due to their appealing feeling on the skin and ease of application [3,4]. Oil in water (O/W) emulsions are usually used in personal care products, which rely on the utilization of hydrophilic polymers as thickeners, rheology modifiers, emulsion stabilizers, emulsifiers and moisturizers [4,5,6].

FucoPol is a high molecular weight exopolysaccharide (EPS) secreted by the bacterium *Enterobacter* A47 (DSM 23139) [7,8,9] composed of fucose, glucose, galactose, glucuronic acid (2.0:1.9:0.9:0.5 relative molar ratio), and acyl groups (acetyl, pyruvyl, and succinyl) that represent up to 12.3 wt.% of FucoPol’s dry mass [10]. FucoPol possesses a→4)-α-L-Fucp-(1→4)-α-L-Fucp-(1→3)-β-D-Glcp(1→trimer backbone. The branches, present at position 3 of the first fucose, are composed of an α-D-4,6-pyruvyl-Galp-(1→4)-β-DGlcAp-(1→3)-α-D-Galp(1→trimer, with two pyruvate caps in the terminal galactose at positions C-4 and C-6 [10,11,12]. This polysaccharide’s properties have been widely reported and include the ability to form viscous solutions with shear-thinning fluid behavior [13], film formation [14,15], emulsion forming and stabilizing capacity [9,16], and bioactivity (antioxidant capacity [17], wound healing ability [12] and photoprotection [18]), which make it a very interesting polysaccharide for biotechnological applications in the field of cosmetics [3,19].

The objective of the present study was to develop emulsions using the bacterial heteropolysaccharide FucoPol as an emulsifying agent, assessing the emulsions’ rheological and textural properties. For that purpose, a preliminary assessment of the emulsion forming and stabilizing capacity of FucoPol for four hydrophobic compounds, at different oil:water (o:w) weight ratios, was conducted. Then, the selected hydrophobic compound (olive oil) was used to prepare emulsions with α-tocopherol. RSM was used to define the optimal concentration ranges for FucoPol, olive oil, and α-tocopherol. The optimized FucoPol-based emulsions were characterized in terms of their rheological textural properties.

## 2. Materials and Methods

### 2.1. Materials

Castor, paraffin, almond, and olive oils were purchased from a local market. Sepigel^®^ 305 was obtained from SEPPIC (Courbevoie, France). α-tocopherol (vitamin E) was acquired from Sigma-Aldrich (Munich, Germany). FucoPol was produced by the bioreactor cultivation of *Enterobacter* A47 (DSM 23139) with glycerol as the sole carbon source as previously described [20], and extracted from the broth by ultrafiltration according to the procedure previously described [16]. FucoPol was composed of fucose (36% mol), glucose (33% mol), galactose (26% mol), and glucuronic acid (5% mol), with a total acyl groups content of 11.1 wt.%. The sample had protein and inorganic salts contents of 13 wt.% and 7.2 wt.%, respectively.

### 2.2. Determination of Surface-Active Properties

FucoPol was dissolved in MilliQ water at concentrations ranging from 0.1 to 20 g/L, and the surface tension of the solutions was determined by the drop pendant method [21] using a Tensiometer (Kruss, Advance, Hamburg, Germany), at room temperature. The critical micelle concentration (CMC) was determined by plotting the surface tension as a function of FucoPol concentration and extrapolating the point where the slope of the curve abruptly changes. The results were expressed as the mean of three solution drops ± standard deviation.

### 2.3. Emulsions’ Preparation

The emulsions were prepared as described by Baptista et al. [16], using castor oil, paraffin oil, almond oil, olive oil as the oil phases, and a FucoPol solution (1.0 or 0.5 wt.%) as the aqueous phase, at o:w weight ratios of 3:2 and 2:3. The emulsification index (EI, %) and the emulsification stability (ES, %) were determined by the following equations [22]:

(1) $$\mathrm{EI}\;=\frac{h_{e}}{h_{T}}\times 100$$

(2) $$\mathrm{ES}\;=\frac{\mathrm{Final}\;\mathrm{EI}}{\mathrm{Initial}\;\mathrm{EI}}\times 100$$

where h_e_ (mm) is the height of the emulsion layer, and h_T_ (mm) is the overall height of the mixture after emulsification. Initial and final EI are the values measured at 24 h and after 720 h (30 days), respectively.

Emulsions with olive oil (o:w weight ratio of 3:2) were also prepared with Sepigel^®^ 305, a commercial emulsifier, at concentrations of 0.1 and 0.5 wt.%. An active ingredient, α-tocopherol, was added to the oil phase at different concentrations (0.0, 2.0, and 5.0 wt.%) and emulsions stabilized with FucoPol or Sepigel^®^ (0.5 and 1.0 wt.%) were prepared with olive oil at an o:w weight ratio of 3:2.

### 2.4. Factorial Design of Experiments

Response surface methodology (RSM) [23] was applied to determine the best formulation to prepare olive oil and α-tocopherol emulsions stabilized with FucoPol. A five-level three-variable central composite design (CCD) was applied, consisting of seventeen runs, with eight factorial points, six axial points, and three central points (Table 1).

The central points are used to determine the experimental error and the reproducibility of the data. The independent variables are coded to have low and high levels of −1 and +1, respectively. The axial points −α and +α were fixed at 1.682 from the central point and make the design rotatable. The mathematical relationship between the independent variables can be approximated by the second-order polynomial model equation:

(3) $$Y=\beta_{0}+\overset{n}{\underset{i=1}{\sum }}\beta_{i}x_{i}+\overset{n}{\underset{i=1}{\sum }}\overset{n}{\underset{j=1}{\sum }}\beta_{ij}x_{i}x_{j}+\overset{n}{\underset{i=1}{\sum }}\beta_{ii}x_{i}^{2}\;$$

where *Y* is the predicted response; *x_i_* are the independent variables (*n* = 3). The parameter *β*_0_ is the model constant; *β_i_* are the linear coefficients; *β_ii_* are the quadratic coefficients, and *β_ij_* are the cross-product coefficients [24]. A full factorial design of experiments was drawn up using the Design-Expert (Design-Expert^®^ software package from Stat-Ease Inc.). The validated model was plotted in a three-dimensional graph and a surface response that corresponds to the best emulsification was generated. Analysis of variance (ANOVA) was used to determine the regression coefficients of individual linear, quadratic, and interaction terms.

### 2.5. Characterization of the Emulsions

#### 2.5.1. Type of Emulsion

The method described by Kavitake et al. [22] was used to determine the type of emulsion. A droplet of the test emulsion was placed onto Whatman™ filter paper (0.2 µm, GE Healthcare Life Sciences, Munich, Germany) and its ability to disperse on the surface was evaluated.

#### 2.5.2. Microscopic Observation

The emulsions were stained with Nile Blue (a lipophilic dye), as described by Martins et al. [25]. Briefly, 10 µL of the emulsion were stained with 1% (*v*/*v*) Nile Blue A (Sigma-Aldrich, Darmstadt, Germany) and observed in a Zeiss Imager D2 epifluorescence microscope (Carl Zeiss, Oberkochen, Germany), with a magnification of 40× through ZEN lite software (Carl Zeiss, Oberkochen, Germany).

#### 2.5.3. Viscoelastic Properties

The emulsions’ rheological properties were studied using an MCR 92 modular compact rheometer (Anton Paar, Graz, Austria), equipped with a PP50/S parallel plate geometry (diameter 50 mm) and a P-PTD 200/AIR Peltier plate to keep the measurement temperature constant at 25 °C. A steady-state flow ramp was used to determine flow curves for shear rates between 0.01 and 1000 s^−1^. The flow curves were fitted to the Cross model [16,26]:

(4) $$\eta =\frac{\eta_{0}}{1+{\left(\tau \left.\dot{\gamma }\right)\right.}^{m}}$$

where *η* is the apparent viscosity (Pa.s), *η*_0_ is the viscosity at zero shear rate (Pa.s), *τ* (s) is the relaxation time (s), and *m* is a dimensionless constant, related to the exponent of pow-er law (*n*) by *m* = 1 − *n* [13,16]. Frequency sweep tests were performed with frequencies ranging from 0.01 to 16 Hz for a constant strain of 0.5% that was well within the linear viscoelastic limit (LVE) evaluated through preliminary amplitude sweep tests.

#### 2.5.4. Texture Analysis

Texture analysis was performed as described by Tafuro et al. [1]. The firmness, consistency, cohesiveness, and adhesivity of the attained formulations were determined using a texture analyser (TMS-Pro, Food Technology Corporation, Sterling, VA, USA) equipped with a 50 N load cell (Mecmesin, Sterling, VA, USA). The sample was placed in a female conic holder and was compressed 11 mm of depth (which represented a sample deformation of around 70%); this procedure was done twice by a male conic probe at a speed of 2 mm/s. The samples’ mechanical parameters were determined from the force–displacement curve: the firmness corresponded to the highest force value attained by the sample during the first compression; the consistency was calculated by the area under the curve of the first compression; the cohesiveness was determined through the ratio of the areas under the curve from the first and the second compressions; and the adhesiveness was determined from the area under the curve from the negative peak attained after the first compression [1,2].

## 3. Results and Discussion

### 3.1. Surface-Active Properties

**Figure 1:**
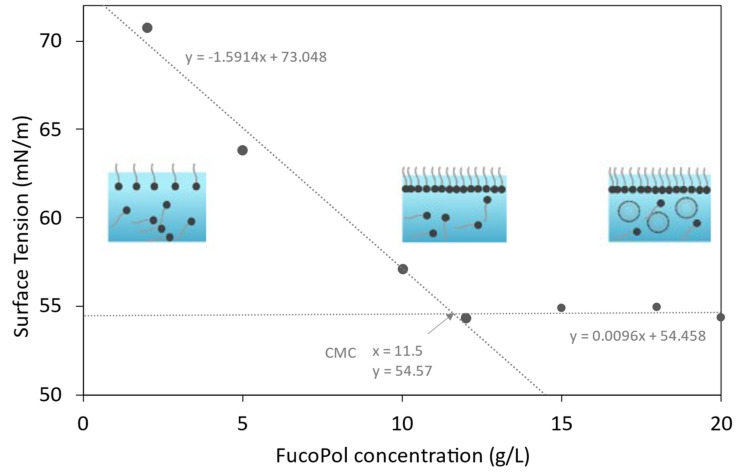
Surface tension of FucoPol solutions at concentrations ranging from 1 to 20 g/L.

Figure 1 shows the equilibrium surface tension as a function of FucoPol concentration. Two distinct regions can be identified: up to around 11.5 g/L, there is a reduction of the surface tension with increasing FucoPol concentration, while above such value the surface tension remains constant irrespective of the biopolymer’s concentration. FucoPol’s critical micelle concentration (CMC) was determined to be approximately 11.5 g/L, given as the point of intersection between the two lines, which correspond to the linear regression of each set of data points [27,28]. This value is considerably higher than those reported for commercial polysaccharides like xanthan and guar gum (approximately 0.15 g/L and 5 g/L, respectively) [29], suggesting a lower thermodynamic stability of the particles system [30]. Nevertheless, FucoPol reduced the surface tension of water from 72 mN/m to 54.6 mN/m at the CMC, a value that is within the range reported for other microbial biosurfactants (34–69 mN/m) [31,32,33]. Moreover, polymeric biosurfactants, despite not significantly lowering the water’s surface tension, are generally more effective in the formation and stabilization of emulsions [31,34].

### 3.2. Emulsion Forming and Stabilizing Capacity of FucoPol

#### 3.2.1. Preparation of FucoPol-Stabilized Emulsions with Different Oils

FucoPol was used to prepare emulsions with four different oils commonly used in cosmetic products’ formulations, namely, castor oil [35,36,37,38], paraffin oil [39,40,41], almond oil [42,43] and olive oil [44,45,46,47,48]. Castor oil is a natural oil that acts as an antimicrobial, anti-inflammatory, antioxidant, wound healing, vasoconstrictive [49] and UV-protective agent [50]. Paraffin oil is a petroleum-based derivative that enables the regulation of viscosity in formulations, possessing protective and lubricating properties which prevent skin dehydration [51]. Almond oil, an abundant macro and micronutrients source, is utilized in cosmetics due to its moisturizing and restructuring properties [42]. Olive oil, composed of squalene, phytosterol, tocopherol, vitamins A and E, and fatty acids (oleic and linoleic acids), is indicated for skin applications due to its acidity and soothing effect [16,39,52].

**Figure 2:**
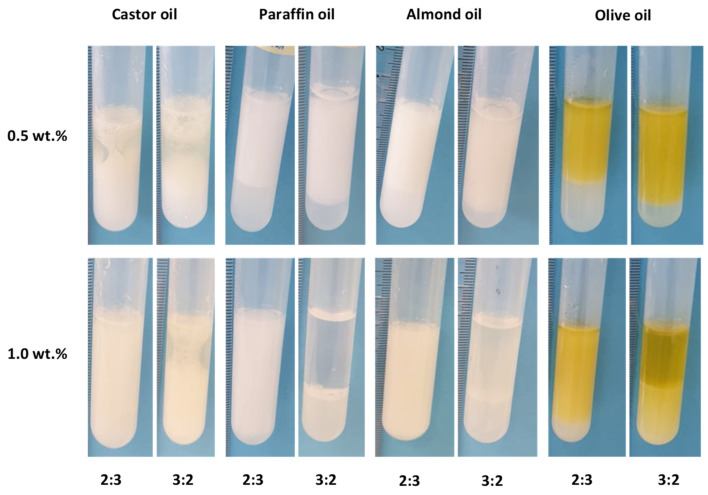
Emulsions prepared with FucoPol (0.5 or 1.0 wt.%) with castor oil, paraffin oil, almond oil, and olive oil, at o:w weight ratios of 2:3 and 3:2.

The assays consisted of mixing the biopolymer, at a concentration of 0.5 or 1.0 wt.%, with each oil, at 2:3 or 3:2 weight ratios. As shown in Figure 2, FucoPol efficiently emulsified all the tested hydrophobic compounds, with EI at 24 h (E24) values above 50% (Table 2), which is the criterion for a good emulsifier [53]. For the 2:3 weight ratio, increasing the concentration of the polymer from 0.5 wt.% to 1.0 wt.% resulted in increased E24 for all the tested oils (Table 2). For the 3:2 weight ratio, on the other hand, this was not observed. In fact, for all tested oils, the E24 value decreased except for castor oil (E24 increased from 56 to 100%). All other oils presented negligible emulsification (Figure 2; Table 2). For 0.5 wt.% of FucoPol, increasing the oil ratio from 2:3 to 3:2 resulted in higher E24, except for castor oil.

#### 3.2.2. Evaluation of Emulsions’ Stability

**Figure 3:**
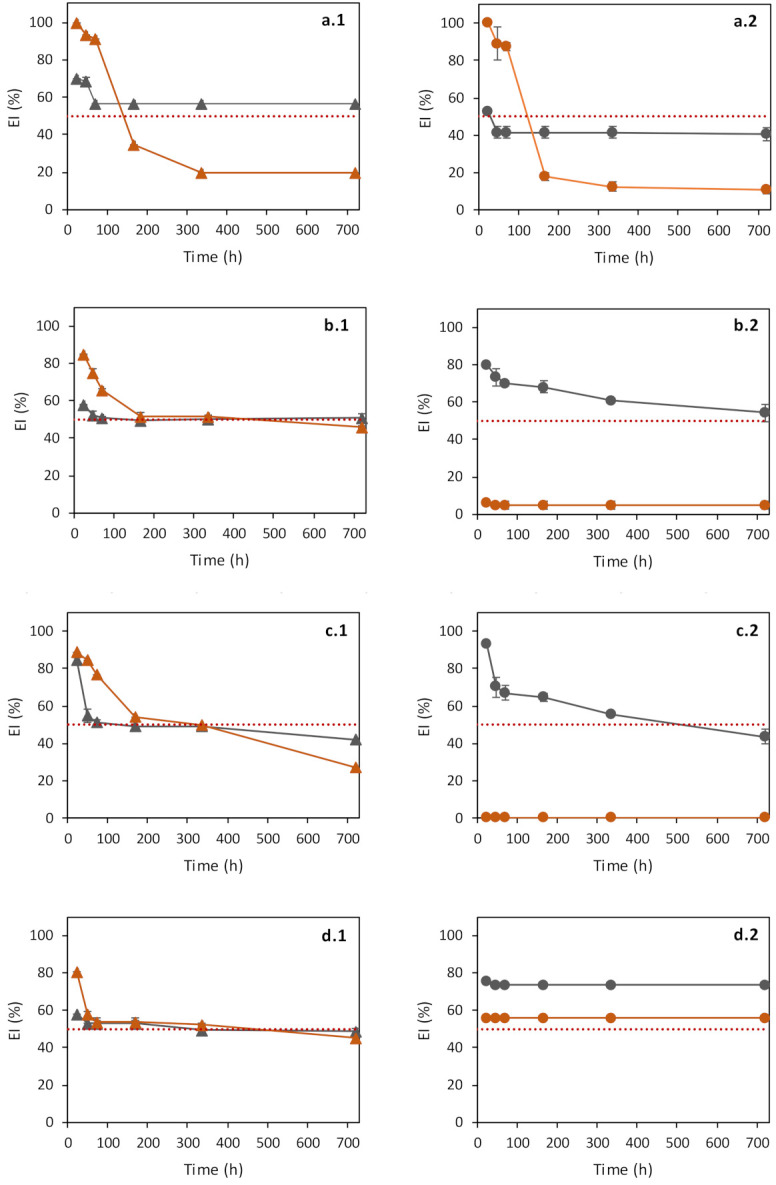
Emulsification index (EI%) overtime for emulsions prepared with FucoPol and different hydrophobic compounds: Castor oil (**a.1**,**a.2**), paraffin oil (**b.1**,**b.2**), almond oil (**c.1**,**c.2**) and olive oil (**d.1**,**d.2**), for FucoPol concentrations of 0.5 wt.% (gray) and 1.0 wt.% (orange), for o:w weight ratios of 2:3 (left) and 3:2 (right). The red dashed line represents EI (%) = 50. (*n* = 3).

Cosmetic applications require that the emulsions have adequate shelf-life, usually up to six months [47,54,55,56]. The stability of the emulsions prepared with FucoPol was evaluated at room temperature, by measuring their EI over a period of 720 h (30 days). As shown in Figure 3, FucoPol emulsion stabilizing capacity depended on the o:w weight ratio, as well as on the tested oil. All FucoPol-stabilized emulsions had no detectable changes in odor or color during the storage period.

The least stable sample was the emulsion prepared with castor oil at an o:w weight ratio of 3:2 and a FucoPol concentration of 1.0 wt.% (Figure 3(a.2)). This sample’s EI dropped from 100% at 24 h to 18% at 7 days, with an overall ES of 11% (Table 2). Nevertheless, the emulsions prepared with castor oil and 0.5 wt.% FucoPol (Figure 3(a.1,a.2)) were stable for both o:w weight ratios, presenting ES values of 81 ± 3% and 77 ± 4%, respectively (Table 2).

Most of the emulsions prepared with paraffin oil and almond oil also showed a significant decrease in their EI during the 720 h shelf-life test (Figure 3(b.1–c.2)) with ES values of 0 to 54% (Table 2). Despite the lower E24 values (56–76%), the olive oil/FucoPol emulsions, for both o:w weight ratios tested, showed higher stability (Figure 3(d.1,d.2)), corresponding to ES values of 85–100% (Table 2). Antunes et al. [42] obtained olive oil/FucoPol emulsions in 2:3 and 3:2 (*v*/*v*) ratios that maintained at least 50% of the initial EI for 9 weeks, which agrees with the results reported in this study.

The results obtained in this study demonstrate that FucoPol is a promising stabilizer for emulsions with any of the tested oils provided the adequate o:w weight ratio and FucoPol concentration are utilized. Castor oil (at the 2:3 weight ratio, 1.0 wt.% FucoPol), paraffin oil (at the 3:2 weight ratio, 0.5 wt.% FucoPol) and olive oil (at the 3:2 weight ratio, 0.5 wt.% FucoPol, and at the 2:3 ratio for either 0.5 or 1.0 wt.% FucoPol) presented high EI and were stable over the 720 h storage period. Given the good results obtained for olive oil and its known biological properties [16,39,52], this oil was chosen for the subsequent studies.

#### 3.2.3. Assaying α-Tocopherol as an Additive to the FucoPol-Stabilized Emulsions

The effect of α-tocopherol, an antioxidant commonly used in cosmetic formulations [57,58], on FucoPol/olive oil emulsions were evaluated by testing different concentrations of this additive on the EI and on the emulsions’ stability. According to the risk profile of tocopherols [59], the maximum concentration of α-tocopherol allowed in cosmetic products is 5 wt.%. Nonetheless, the α-tocopherol concentration in the skin care cosmetics below 0.2% is sufficient to protect lipids against peroxidation [60]. Therefore, α-tocopherol at concentrations of 2.0 and 5.0 wt.% were selected for testing as an additive in FucoPol/olive oil emulsions.

**Figure 4:**
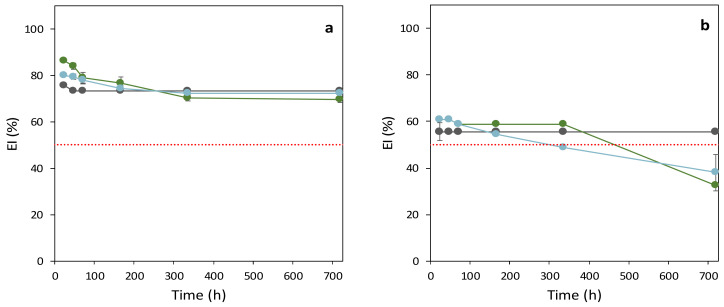
Emulsification index (EI%) over time for the FucoPol-stabilized emulsions with olive oil and α-tocopherol, at the 3:2 ratio: FucoPol (0.5 wt.% (**a**); 1.0 wt.%, (**b**)); α-tocopherol: 0 wt.%, gray; 2.0 wt.%, green; 5.0 wt.%, blue. The red dashed line represents EI = 50%. (*n* = 3).

As shown in Table 3, the addition of α-tocopherol led to an increase in the E24 values for both FucoPol concentrations tested. Compared to the samples with no α-tocopherol that had E24 values of 76% and 56%, for FucoPol concentrations of 0.5 and 1.0 wt.%, respectively (Table 3), the addition of α-tocopherol resulted in higher E24 (80–86% and 61%, respectively). However, the resulting emulsions were less stable, especially for those prepared with 1.0 wt.% FucoPol that had overall ES of 61%, compared to 96% for the sample with no α-tocopherol (Figure 4, Table 3). For the emulsions prepared with 0.5 wt.% FucoPol, the ES was 82% and 90%, for 2.0 and 5.0 wt.% α-tocopherol, respectively (Table 3). Despite the observed ES reduction, the sample containing 5.0 wt.% α-tocopherol had an ES of 90 at 720 h, identical to the sample with no additive (Table 3).

#### 3.2.4. Comparison with Sepigel^®^ 305

The commercial emulsifier agent Sepigel^®^ 305 (also known as Farcosgel) was used to prepare emulsions with olive oil (o:w weight ratio of 3:2), with and without α-tocopherol, and the results were compared to those of FucoPol’s emulsions (Table 3). Sepigel^®^ 305 is a synthetic hydrophilic polymer used in cosmetics to provide increased viscosity and stability to the formulation [61]. Sepigel^®^ 305 is composed by a blend of polyacrylamide (10–20%), C13–14 Isoparaffin (1–5%), and Laureth-7 (1–5%). In its composition, each compound presents a specific function: the pre-neutralized polyacrylamide polymer is contained within an emulsion, where isoparaffin forms an oily phase and laureth-7 acts as a surfactant [61,62,63]. The comparison between Sepigel^®^ 305 and FucoPol aims to discuss the possibility to replace a widely used chemical agent with a naturally produced polymer in cosmetic formulations. Interestingly, no emulsification was observed for the 0.5 wt.% Sepigel^®^ samples and an EI of 49 ± 0% was obtained for 1.0 wt.%, which was, nevertheless, stable for the 720 h shelf-life periods assay. The addition of α-tocopherol had a negative impact, and no emulsification was observed for any additive concentration. According to Anchisi et al. [56], Sepigel^®^ 305 at concentrations of 1.5–7%, with an oil phase consisting mainly of a fluid oil, resulted in good emulsification for O/W skin creams. Other studies have reported the development of stable emulsions containing this polymer at concentrations higher than 2 wt.% [61,62,63,64]. At lower concentrations (<1.5 wt.%), the synthetic hydrophilic polymer was able to stabilize O/W formulations only with the addition of different emulsifying ingredients and emulsion stabilizers [65,66]. These results show the ability of the natural polymer FucoPol to emulsify without the addition of other agents at low concentrations, which becomes an advantage compared to the synthetic polymer Sepigel^®^ 305.

### 3.3. Emulsification Optimization by Response Surface Methodology

#### 3.3.1. Response Analysis

Table 4 shows the data for the 17 runs of the CCD. Results show that the emulsification after 24 h ranged from 0.0 to 97.8%. Good emulsification index (E24 > 95%) was obtained in runs 1, 5, 11, 12, 14, 16 and 17, for which FucoPol concentration was 0.8–1.3 wt.%, and the olive oil content was 20–30 wt.%, irrespective of the α-tocopherol content that varied from 0–5 wt.%. These results suggest that α-tocopherol concentration has little effect on the E24. Outside those FucoPol and olive oil concentration ranges, E24 of 30.4–78.3% were attained. As expected, no emulsification was obtained in run 15 due to the absence of the bioemulsifier. Moreover, there was also no emulsification for runs 2, 6 and 10.

#### 3.3.2. RSM Modelling

RSM methodology was used to evaluate the effect of each ingredient (FucoPol, olive oil and α-tocopherol) on the E24 of the emulsions, as well as the combined effect of the variables. ANOVA was used to define the working ranges for each variable resulting in the highest E24 values. The statistical analysis (Table 5) shows that the proposed model was adequate [67]. The quadratic model was found to be significant (*f*-value = 18.51 and *p*-value = 0.001), and it was supported by an insignificant lack-of-fit (*p* = 0.634) toward the response (E24). There is only 0.10% chance that a “Model F-Value” could occur due to noise, meaning that the greater *f*-value from unity explains adequately the variation of the data around its mean; in addition, the estimated factor effects are real [68,69]. The R^2^ (0.965) was in reasonable agreement with the adjusted R^2^ (0.913). The adjusted coefficient of determination indicated that 91.31% of the variability in the response could be explained by the model. Hence, the quadratic model is an accurate representation of the actual relationships between the response and the variables. The observed precision of 12.19 indicates an adequate signal (ratio > 4 is desirable). The statistical analysis indicates that the proposed model was adequate to predict the ingredients’ concentrations to obtain stable emulsions (E24 > 50%) [53].

**Figure 5:**
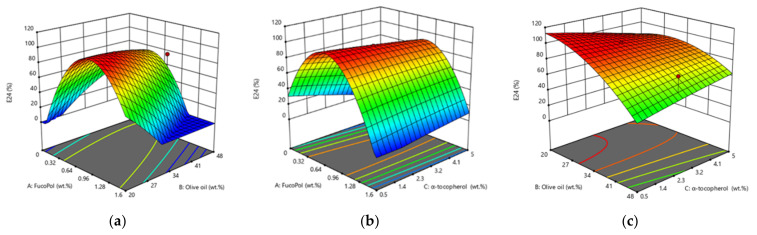
Three-dimensional response surface plot showing the interactive effects of different components on the O/W emulsion. (**a**) FucoPol and Olive oil (wt.%) with α-tocopherol fixed at 2.5 wt.%, (**b**) FucoPol and α-tocopherol (wt.%) with olive oil fixed at 30 wt.%, (**c**) olive oil and and α-tocopherol (wt.%) with FucoPol fixed at 0.8 wt.%.

The response of the RSM was shown as three-dimensional surface graphs (Figure 5, and contour plots resulting in an infinite number of combinations of the FucoPol, olive oil, and α-tocopherol. The result suggests that FucoPol concentrations between 0.8–1.3 wt.% and olive oil concentrations between 20–30 wt.% reach E24 values above 95.6%. Moreover, α-tocopherol does not appear to influence the E24 value (Figure 5). Figure 5a shows an inversely proportional interaction between FucoPol and olive oil, whereby E24 value increases with the increase in FucoPol concentration and decrease in olive oil concentration. Figure 5b corroborates the observed inverse proportionality between FucoPol and olive oil concentrations. Lastly, Figure 5c shows higher E24 values for olive oil between 25–30 wt.%.

Based on Figure 5, increasing the concentration of FucoPol resulted in emulsions more stable against coalescence, avoiding emulsion phase separation [70]. This is due to FucoPol’s ability to allow a specific texture (of increased viscosity) to the formulation and to decrease elasticity-driven creaming of the droplets [3]. FucoPol concentration and olive oil concentration have inversely proportional effects, as shown by the *p*-value < 0.05 (Table 5). In this case, linear (B), interaction (AB) and quadratic (A^2^) are significant model terms on E24 including a positive linear effect (*p* = 0.004) of olive oil and a quadratic effect (*p* = 0.0001) of FucoPol, interacting with himself on the response [67]. This result agrees with results obtained for bacterial cellulose, in which emulsions became more stable as the concentration increased, reaching 1 wt.% [71]. In contrast, for xanthan gum concentrations of 0.12% and 0.2%, emulsions became more stable with 50 wt.% of oil [72,73,74].

Based on the results obtained in the CCD, two FucoPol-stabilized emulsions were prepared: F1 that comprised 1.0 wt.% FucoPol, 25 wt.% olive oil and 0.5 wt.% α-tocopherol; and F2 that comprised 0.7 wt.% FucoPol, 25 wt.% olive oil and 2.0 wt.% α-tocopherol. F1 and F2 yielded E24 values of 98.0 ± 0.40% and 84.7 ± 0.0%, respectively.

### 3.4. Characterization of the FucoPol-Stabilized Emulsions

#### 3.4.1. Type of Emulsion

**Figure 6:**
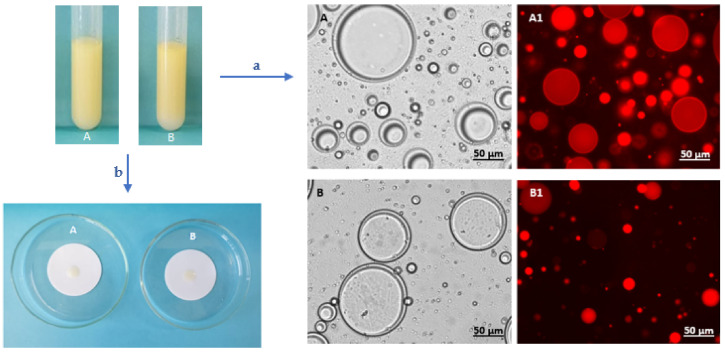
(**a**) Optical microscopic (40×) images of F1 (**A**) and F2 (**B**) emulsions; contrast phase and fluorescence after Nile Blue A staining (**A1**, **B1**, respectively); (**b**) emulsion determination test by filter paper wetting.

The microscopic observation (Figure 6a) of the emulsions showed compartmentalized structures characteristic of O/W emulsions, consisting of dispersed oil droplets in the aqueous phase [75,76]. Furthermore, in the emulsion determination test (Figure 6b), the emulsions’ droplets rapidly dispersed on the filter paper, thus confirming their O/W nature [22,77,78]. O/W emulsions represent nearly 65% of the total emulsified products available in the cosmetic industry market due to their sensorial properties [79,80] and are present in several products such as creams and lotions [80,81]. W/O emulsions are commonly used in waterproof products by providing higher hydration to emulsions [80]. However, these emulsions usually are responsible for an oily sensation on the skin, which enhances the consumer preference for O/W emulsified products [82].

#### 3.4.2. Viscolelastic Properties

**Figure 7:**
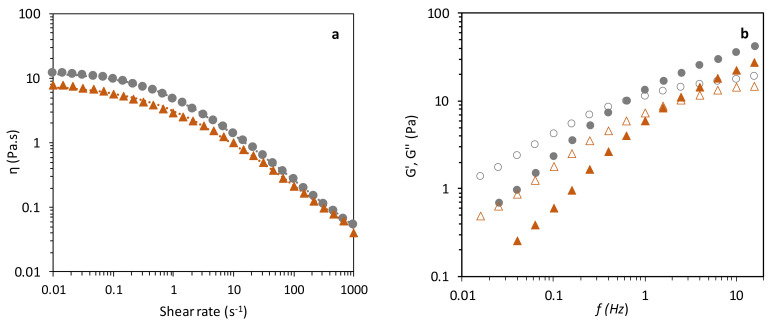
Rheological profile analysis of FucoPol formulations F1 (circles) and F2 (triangles): (**a**) viscosity curves as a function of the shear rate, flow curves fitted with Cross model (*n* = 3); (**b**) elastic G’ (closed) and viscous G” (open) moduli in the function of frequency.

As shown in Figure 7a, both samples presented a shear-thinning behaviour, as the viscosity progressively decreased under increasing shear rates, in agreement with previous studies that reported the same behaviour for FucoPol/olive oil emulsions [16]. This effect is observed when a spherical shape is detangled by polymer chains and the droplets begin to deform, forming an ellipsoidal shape. Moreover, layer formation, due to aggregate breaking into elemental constituents, is concurring with the shear plane, decreasing the overall flow resistance [1,2,83,84]. This shear-thinning behaviour was observed for emulsions stabilized by other polysaccharides, such as xanthan gum and guar gum [83,85]. Nevertheless, slight differences are noticed between samples, namely, a lower apparent viscosity for the emulsion F2 (Figure 7a, triangles) compared to emulsion F1 (Figure 7a, circles), which was probably due to the lower polymer concentration in F2. FucoPol increased viscosity in the water phase leads to decreased droplets’ mobility and collision numbers, which can explain the observed behaviour [86].

A non-Newtonian mathematical model, the Cross model, was fitted to the experimental results (Figure 7a) with the resulting parameters given in Table 6. The highest ƞ_0_ value was observed for emulsion F1 (13.92 ± 2.36 Pa.s), but the τ fitting parameter was similar for both emulsions. The emulsions had an identical degree of shear-thinning as shown by the similar values of m (0.74 ± 0.00 and 0.68 ± 0.01) [87].

The mechanical spectra (Figure 7b) of the two FucoPol-stabilized emulsions showed higher loss modulus (G″) than storage modulus (G′), indicating a liquid-like behaviour [16], with FucoPol viscosity being the dominant property influencing the emulsions’ stability [54,88]. The mechanical spectra for the two emulsions are quite similar, with G″ increasing at a higher rate than G′, with the crossover of dynamic moduli being perceived at a lower frequency (0.6 Hz) for emulsion F1 than for emulsion F2 (1.6 Hz). This indicates that, for emulsion F1, higher viscosity translates into lower energy storage threshold, featuring a G′ G″ crossover at a lower frequency [16,89]. After the crossover point, increasing the frequency displays a solid-like behaviour for both emulsions (G′ > G″) [54,90].

#### 3.4.3. Textural Assessment

As shown in Table 6, the FucoPol-stabilized emulsions F1 and F2 had the same firmness (0.074 N) when perforated 11 mm with a conic probe. The consistency of emulsion F1 was 0.088 mJ, while that of sample F2 was 0.055 mJ. Studies showed that the firmness and energy required to deform a sample are related to the sample’s spreadability: high firmness and consistency values indicate a less spreadable sample, whilst lower consistency and firmness values indicate a more spreadable sample [1,2]. Hence, these results show that both samples are very spreadable, presenting low firmness and consistency values. Moreover, emulsion F2 was more spreadable than sample F1 (Table 6). The spreadability (skin cover capacity over time) is crucial in cosmetic emulsion development being a decisive factor for consumers’ approval of products [91]. While both samples showed some adhesivity, emulsion F1 (0.156 mJ) seemed to be more adhesive than F2 (0.129 mJ). When a formulation is spread, verifying the material’s uniform scattering throughout the applied surface is pivotal to avoid the active substance’s accumulation or dissipation and to insure the correct utilization of the formulation [1,2]. Therefore, the cohesiveness was also an important parameter to be observed. Given this, emulsion F1 (0.748) is more cohesive than emulsion F2 (0.688), which concludes that sample F1 has higher firmness, adhesiveness and cohesiveness but is less spreadable than sample F2. These results are concordant with *η*_0_ (Pa.s) values (Table 6), where emulsion F1 exhibited higher apparent viscosity (13.92 ± 2.36 Pa.s) than F2 (7.59 ± 0.04 Pa.s).

#### 3.4.4. Comparative Analysis of the FucoPol-Stabilized Emulsions

F2 emulsion had a slightly higher spreadability value [91], a feature of interest for cosmetic and pharmaceutical applications. Furthermore, at high shear rates (e.g., 1000 s^−1^, which is representative of a skin spreading process [91]), both F1 and F2 emulsions displayed analogous viscosity (0.05 Pa.s and 0.04 Pa.s, respectively). Such characteristics are found in lotions or light creams [80,82], thus confirming the potential of FucoPol for the development of skin care cosmetic products.

## 4. Conclusions

This study demonstrated the ability of the bacterial polysaccharide FucoPol to emulsify olive oil and α-tocopherol, outperforming the commercial emulsifier Sepigel^®^. The resulting O/W emulsions had good viscosity and spreadability, which substantiates its relevance in the development of cosmetic applications. The emulsion textural properties can be modulated by using different FucoPol and α-tocopherol contents, thus yielding formulations suitable for use in different skin-care products. The intrinsic antioxidant capacity of FucoPol adds to that of α-tocopherol, which, together with FucoPol’s wound-healing ability, render this natural polysaccharide as a valuable biomaterial for cosmetic formulations’ development.

## Figures and Tables

**Table 1:** Independent variables and their levels used in the response surface design.

Independent Variables	Coded Variable	Factor Level				
		−α	−1	0	1	α
FucoPol (wt.%)	A	0.00	0.30	0.80	1.30	1.64
Olive oil (wt.%)	B	13.18	20.00	30.00	40.00	46.82
α-tocopherol (wt.%)	C	0.00	1.00	2.50	4.00	5.02

**Table 2:** Emulsification activity measured at 24 h (E24) and emulsions’ stability (ES) for the emulsions stabilized with FucoPol. Data are shown as the average ± standard deviation (SD) (*n* = 3).

Oil	FucoPol	E24 (%)		ES (%)	
		2:3	3:2	2:3	3:2
Castor oil	0.5%	70 ± 3	53 ± 4	81 ± 3	77 ± 4
	1.0%	100 ± 0	100 ± 0	20 ± 0	11 ± 0
Paraffin oil	0.5%	58 ± 2	80 ± 4	87 ± 5	68 ± 6
	1.0%	85 ± 2	6 ± 0	54 ± 1	78 ± 0
Almond oil	0.5%	84 ± 4	93 ± 6	50 ± 3	47 ± 6
	1.0%	89 ± 0	0 ± 0	31 ± 6	0 ± 0
Olive oil	0.5%	58 ± 0	76 ± 0	85 ± 2	97 ± 0
	1.0%	81 ± 1	56 ± 0	56 ± 1	100 ± 0

**Table 3:** Emulsification activity at 24 h (E24) and emulsions’ stability over a period of 720 h, for the emulsion prepared with FucoPol, olive oil and α-tocopherol, at 3:2 (*w*/*w*) ratio. Data are shown as the average ± standard deviation (SD) (*n* = 3).

Emulsifier	α-Tocopherol (wt.%)	0.5 wt.% Emulsifier		1.0 wt.% Emulsifier	
		E24 (%)	ES (%)	E24 (%)	ES (%)
FucoPol	0	76 ± 0	87 ± 3	56 ± 0	96 ± 0
	2.0	86 ± 1	82 ± 1	61 ± 0	53 ± 5
	5.0	80 ± 1	90 ± 1	61 ± 0	63 ± 12
Sepigel	0	0 ± 0	-	49 ± 0	100 ± 0
	2.0	0 ± 0	-	0 ± 0	-
	5.0	0 ± 0	-	0 ± 0	-

**Table 4:** Central composite design (CCD) with studied variables (A: FucoPol, B: Olive oil, C: α-tocopherol), experiment and theoretically predicted values E24.

Run	FucoPol, A (wt.%)	Olive Oil, B (wt.%)	α-Tocopherol, C (wt.%)	E24 (%)	
				Actual Value	Predicted Value
1	0.8	30	2.5	97.8	97.1
2	1.6	30	2.5	0.0	3.0
3	0.3	20	4.0	30.4	26.8
4	0.8	46.8	2.5	78.3	57.3
5	0.8	30	0.0	95.7	100
6	1.3	40	4.0	0.0	19.5
7	0.3	20	1.0	76.1	56.6
8	0.3	40	4.0	69.6	72.7
9	0.8	13.2	2.5	73.9	95.0
10	1.3	40	1.0	0.0	3.6
11	0.8	30	5.0	95.7	90.0
12	0.8	30	2.5	97.7	97.1
13	0.3	40	1.0	69.6	78.7
14	0.8	30	2.5	97.6	97.1
15	0.0	30	2.5	0.0	15.3
16	1.3	20	4.0	95.6	87.0
17	1.3	20	1.0	97.8	94.6

E24 predicted value = 97.07 − 3.82A − 11.24B − 3.5C − 28.26AB + 5.43AC + 5.98BC − 34.31A2 − 7.41B2 − 0.4880C2.

**Table 5:** ANOVA for response surface quadratic model. (SS)—Sum of Squares shows the variance of values; (MS)—Mean Square is the arithmetic mean of the squared differences; *p*-value < 0.05 indicate model terms are significant.

Source	SS	MS	*f*-Value	*p*-Value	Significance
Model	24,439.63	2715.51	18.51	0.001	Significant
A: FucoPol	199.37	199.37	1.360	0.287	
B: Olive oil	2854.49	2854.49	19.46	0.004	
C: α-tocopherol	167.58	167.58	1.140	0.326	
AB	6390.15	6390.15	43.56	0.001	
AC	236.31	236.31	1.610	0.251	
BC	286.08	286.08	1.950	0.212	
A^2^	14,317.43	14,317.43	97.60	0.0001	
B^2^	0.0002	0.0002	1.4 × 10^−6^	0.999	
C^2^	72.96	72.96	0.497	0.507	
Lack of Fit	582.37	145.59	0.766	0.634	Not significant
R^2^	0.965				
R^2^ adjusted	0.913				
R^2^ predicted	0.622				
Adequate precision	12.19				

**Table 6:** Cross model parameters estimated for formulations samples: *η*_0_—apparent viscosity of the second Newtonian plateau (Pa·s); *τ*—relaxation time (s); *m*—dimensionless constant; Data are shown as the average ± standard deviation (SD) (*n* = 3); and textural parameters.

Emulsion	Cross Model Parameters			Textural Parameters			
	*η*_0_ (Pa.s)	*τ* (s)	*m*	Firmness (N)	Consistency (mJ)	Adhesiveness (mJ)	Cohesiveness
F1	13.92 ± 2.36	1.64 ± 0.13	0.74 ± 0.00	0.074	0.088	0.156	0.748
F2	7.59 ± 0.04	1.72 ± 0.04	0.68 ± 0.01	0.074	0.055	0.129	0.688

RE = $\overset{n}{\underset{i=1}{\sum }}\left(\left|\mathrm{xeI}–,i-\;\mathrm{xcalc},i\right|/\mathrm{xexp}\right)/n\;$ is between 0.011 and 0.019.

## Data Availability

Data will be available upon request.
